# Clinicopathological and prognostic significance of LKB1 expression in gastric cancer: a systematic review and meta-analysis

**DOI:** 10.1038/s41598-023-36239-5

**Published:** 2023-06-01

**Authors:** Guojiang Tan, Baiying Liu

**Affiliations:** Department of Gastrointestinal Surgery, The Third XiangYa Hospital of Central South University, Changsha, China

**Keywords:** Cancer, Biomarkers

## Abstract

Many studies report Liver kinase B1 (LKB1) plays a critical role in gastric cancer (GC). However, the relationship between LKB1 and the clinicopathological parameters of GC patients remains controversial. This meta-analysis aimed to investigate the above question and re-evaluate the prognostic significance of LKB1 in GC patients. We searched PubMed, Web of Science, Cochrane Library, Google Scholar, CNKI, and Wan Fang to identify relevant studies published before April 20, 2023. After careful screening, 11 studies involving 1767 patients were included. We found that LKB1 expression was significantly related to tumor size (OR 0.515; 95% CI 0.316–0.839; *P* < 0.01), differentiation (OR 0.643; 95% CI 0.521–0.794; *P* < 0.001), depth of invasion (OR 0.397; 95% CI 0.319–0.494; *P* < 0.001), lymph node metastasis (OR 0.487; 95% CI 0.397–0.598; *P* = 0.01), and TNM stage (OR 0.362; 95% CI 0.293–0.447; *P* = 0.006). However, LKB1 was unrelated to gender and age (*P* > 0.05). Moreover, low LKB1 expression was significant correlate with overall survival (OS) (HR = 1.59; 95% CI 1.29–1.96; *P* < 0.001). In conclusion, LKB1 expression is related to tumor size, differentiation, depth of invasion, lymph node metastasis, and TNM stage, and low LKB1 expression can predict a poor prognosis. LKB1 is a potentially valuable prognosis signature and therapeutic target in GC patients.

## Introduction

Gastric cancer (GC) is one of the most common digestive cancer, ranking fifth in the frequency of cancer incidence and fourth in cancer-related death worldwide^1^. Furthermore, GC shows a higher incidence and mortality in East Asia, especially in China^2^. GC is the third most frequently diagnosed in China and the third cause of cancer-related death^3^. According to the previous study, most patients with GC have no apparent early-stage symptoms, with approximately 50% of patients diagnosed at an advanced stage^4^. Many strategies used for GC treatment include surgery, chemotherapy, radiation therapy, targeted therapy, and immune therapy^5^. However, the 5-year overall survival (OS) rate for GC remains low (32.4%) worldwide^6^. Consequently, early detection and effective treatment of GC are critical.

Liver kinase B1 (LKB1), also known as STK 11 (serine–threonine kinase 11), is a serine–threonine kinase that activates adenosine monophosphate-activated protein kinase (AMPK) by reducing intracellular adenosine triphosphate^7^. It is essential in cellular functions, including cell cycle progression, metabolism, differentiation and polarity^8^, lipid cholesterol and glucose metabolism^9^. Nowadays, the role of LKB1 plays in cancer has increasingly become a focus. LKB1 is considered as a protective factor in lung cancer, controlling its initiation, differentiation and metastasis by repressing metastasis-promoting genes, such as NEDD9, VEGFC and CD24^10^. Moreover, LKB1 expression plays a vital role in many cancers, such as hepatocellular carcinoma^11^, prostate cancer^12^, and breast cancer^13^. As LKB1 plays an essential role as a tumor suppressor, many studies were conducted in GC to explore the association of LKB1 expression with clinicopathological features or prognosis; however, the results remain controversial. Yin et al. found high LKB1 expression correlated with GC differentiation^14^. While Sun et al. reported that high LKB1 expression is not connected with differentiation in GC^15^. Hu et al. suggest that high LKB1 expression is associated with lymph node metastasis^16^. However, Ma et al. demonstrated that high LKB1 expression in GC is not associated with lymph node metastasis^17^. Therefore, we conducted this meta-analysis to better inform clinicians of the relationship between LKB1 expression and the clinicopathological features, as well as the predictive outcomes of GC patients.

## Methods

### Literature retrieval strategy

By April 20, 2023, relevant literature had been retrieved from the following databases: PubMed; Web of Science; Google Scholar; Cochrane Library; CNKI and Wan Fang. The search terms used for screening were as follows: (“Gastric cancer” or “Stomach cancer” or “Gastric neoplasm” or “Stomach neoplasm”) and (“STK 11” or “Serine-Threonine Kinase 11” or “LKB1” or “Liver kinase B1”). The literature was selected following PRISMA guidelines by two reviewers independently. Any conflicting opinion was solved by discussion and re-evaluation.

### Inclusion and exclusion criteria

Studies met the following criteria were included: (1) retrospective, clinical design; (2) use of immunohistochemistry (IHC) to detect LKB1 expression in GC specimens; (3) focus on the relationship between LKB1 expression and the clinicopathological features of patients with GC; and (4) sufficient data for the calculation of the odds ratio (OR) and 95% confidence interval (CI).

The exclusion criteria were as follows: (1) duplicate publications; (2) review, letter, or case report; (3) animal studies; (4) not using IHC to detect LKB1 expression; (5) studies lack clinicopathological data or cell assay only.

### Quality assessment

Two reviewers independently assessed the quality of the included studies using the standard Newcastle–Ottawa Scale (NOS), with scores ranging from 0 to 9^18^. The NOS contains the following scoring items: Selection, Comparability, and Outcome. NOS scores above 6 are considered high quality^18^. The studies included in this meta-analysis range from 7 to 9.

### Data extraction

Two researchers acquired the relevant data by independently reading the studies’ full texts and extracting the following information: (1) name of the first author, publication year, publication journal, country, research type, and sample capacity; (2) tumor histology, detective method, cut-off value, antibody information; (3) age, gender, adjuvant therapy, endpoint event, follow-up time; (4) tumor differentiation, depth of invasion, tumor size, lymph node metastasis, tumor stage and the overall survival (OS) data of patients. All data were cross-examined by two investigators. Disagreements were resolved by a third investigator. We contact the corresponding author to gather accurate data if the study information needs to be completed or cleared. If the HR and CIs cannot be extracted from the article directly, we use Engauge Digitizer software 4.1 to obtain data from Kaplan–Meier curves and calculate HR and 95% CIs^19^.

### Statistical analysis

The odds ratio (OR) was used to evaluate the Binary variable, which determines whether an exposure factor is a protective or risk factor for positive events. An OR > 1 indicates that the exposure factor was a risk factor for positive events. An OR equal to 1 means no statistical significance. An OR < 1 suggests exposure is a protective factor for positive events^18^. The hazard ratio (HR) was used to evaluate the OS of GC patients. A 95% confidence interval (CI) was used to estimate the OR and HR. In this study, the Q test and I^2^ value were used to assess the heterogeneity of each survey. I^2^ values < 25%, 25–50%, and > 50% indicated mild, moderate, and high heterogeneity, respectively^20^. The fixed-effects model calculates the summary estimate if the Q statistic *P* value is more than 0.1^21^. Otherwise, the random-effects model was used to estimate. Additionally, sensitivity analysis was conducted to confirm the robustness of this meta-analysis. Publication bias was assessed by Begg’s and Egger’s tests. STATA 12 (StataCorp, College Station, TX, USA) was used to analyze the data in the present meta-analysis, with statistical significance set at *P* < 0.05.

## Results

### Search results and study characteristics

**Figure 1:**
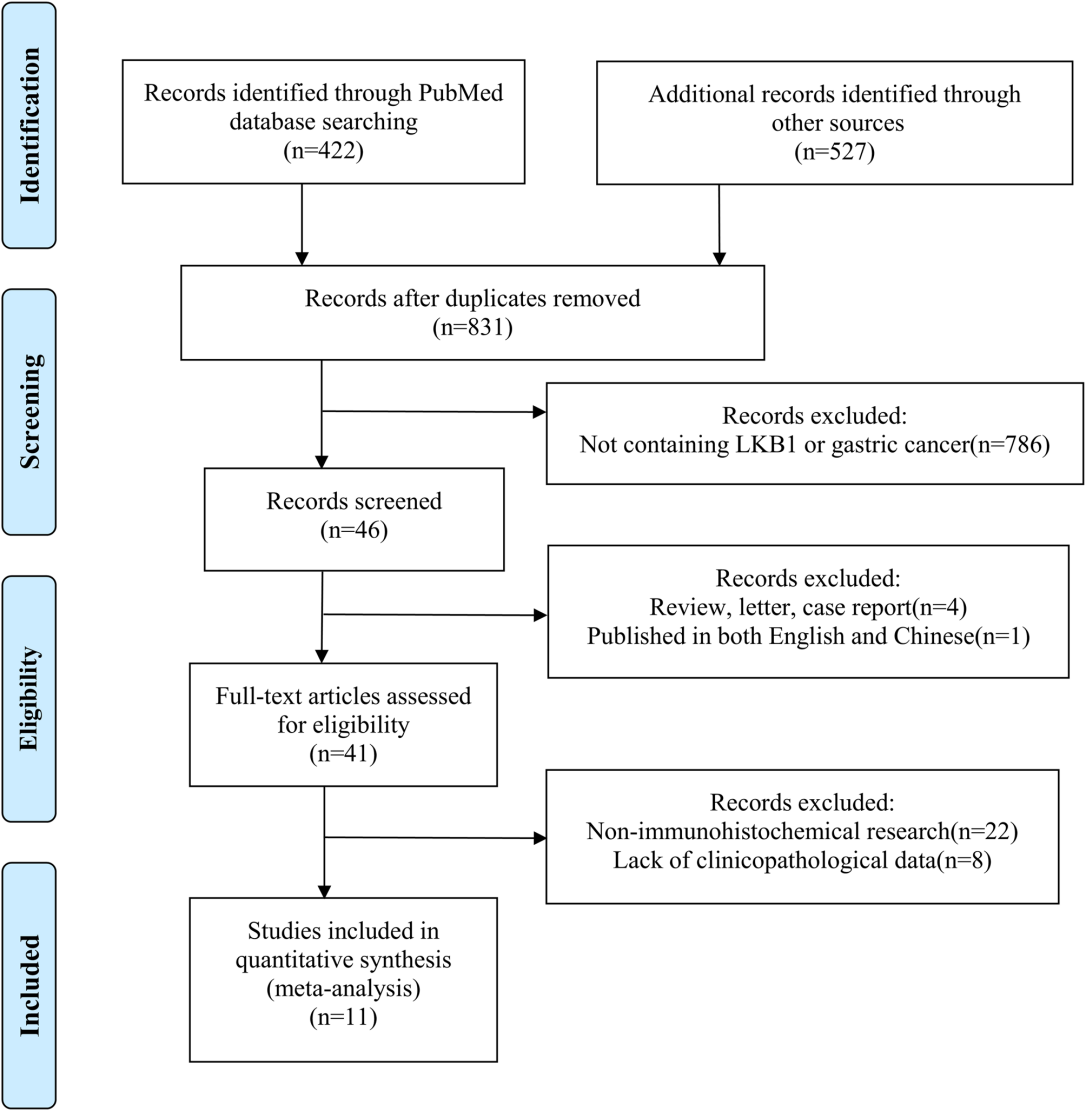
Flowchart for selection of 11 articles.

The initially retrieved literature from the databases above is 949. Among them, 422 studies were filtered from PubMed using the retrieval methods mentioned above, and 527 were selected from other databases. After excluding duplicate and irrelevant studies that did not explore the relationship between LKB1 and GC, 46 studies were enrolled. By browsing the study abstract, 41 studies were identified after excluding reviews, letters, case reports, and one study published in English and Chinese. After reading the complete text, 30 studies were excluded owing to a lack of clinicopathological data and the use of non-IHC methods. Finally, 11 studies were included in our analysis based on the predefined criteria, and the selection process is detailed in Fig. 1, and the detailed selection process in PubMed is shown in Figure S1. Among the 11 studies, 10 were from China, and one was from Japan, with sample sizes ranging from 60 to 708. Overall, the meta-analysis included 1767 patients with GC^4,15,16,22–29^, and the detailed characteristics are shown in Table 1, and the detailed NOS assessment is shown in Table S1.

**Table 1:** Main characteristics of the eligible studies.

Author	Year/country	Case	Gender(M/F)	Antibody source of LKB1	Dilution	Detection/method	Cut-off point (high/low)	Follow up (months)	Endpoints	NOS Score
Li WW	2019/China	150	112/38	Omnimabs	1:1000	IHC	High: Score ≥ 3 Low: Score < 3	60	OS	8
Jiang SS	2016/China	63	48/15	Santa Cruz Biotechnology	1:250	IHC	NA	NA	NA	9
Hu M	2019/China	107	81/26	OmnimAbs	1:1000	IHC	High: Score ≥ 2 Low: Score < 2	60	DFS	8
Ma LG	2016/China	109	71/38	Sigma-Aldrich	1:500	IHC	High: Score > 1 Low: Score≤ 1	100	OS	7
Sun JJ	2016/China	155	121/34	Cell Signaling Technology	NA	IHC	High: staining area > 10% Low: staining area < 10%	80	OS	8
Nishimura S	2020/Japan	708	481/227	Santa Cruz	NA	IHC	High: staining area > 10% Low: staining area < 10%	60 s	OS	7
Zhao ZG	2019/China	120	68/52	Shanghai Changdao Biological Co. LTD	NA	IHC	High: Score ≥ 8 Low: Score < 8	60	OS	8
Yin M	2017/China	110	77/33	Proteintech	1:500	IHC	High: Score ≥ 4 Low: Score < 4	NA	OS	8
Li LY	2015/China	70	39/31	Santa Cruz	NA	IHC	High: Score ≥ 5 Low: Score < 5	NA	NA	7
Huang Y	2014/China	115	73/42	Abcam	1:100	IHC	High: staining area > 10% Low: staining area < 10%	80	OS	8
Ge MZ	2010/China	60	39/21	Abcam	1:100	IHC	High: Score ≥ 2 Low: Score < 2	NA	NA	7

### Relationship between LKB1 expression and gender

**Figure 2:**
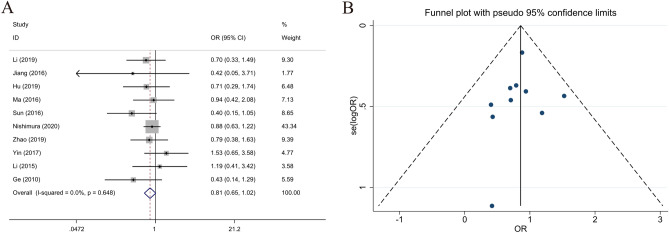
Forest plot (fixed-effects model) and funnel plot for publication bias test for the association between LKB1 expression and gender (**A**,**B**) of patients with gastric cancer.

Gender data on 1137 males and 515 females were collected from 10 studies. Analysis results revealed no relationship between high LKB1 expression and gender in patients with GC (male vs. female, OR 0.815; 95% CI 0.654–1.016; *P* = 0.068, I^2^ = 0.0%) (Fig. 2).

### Relationship between LKB1 expression and age

**Figure 3:**
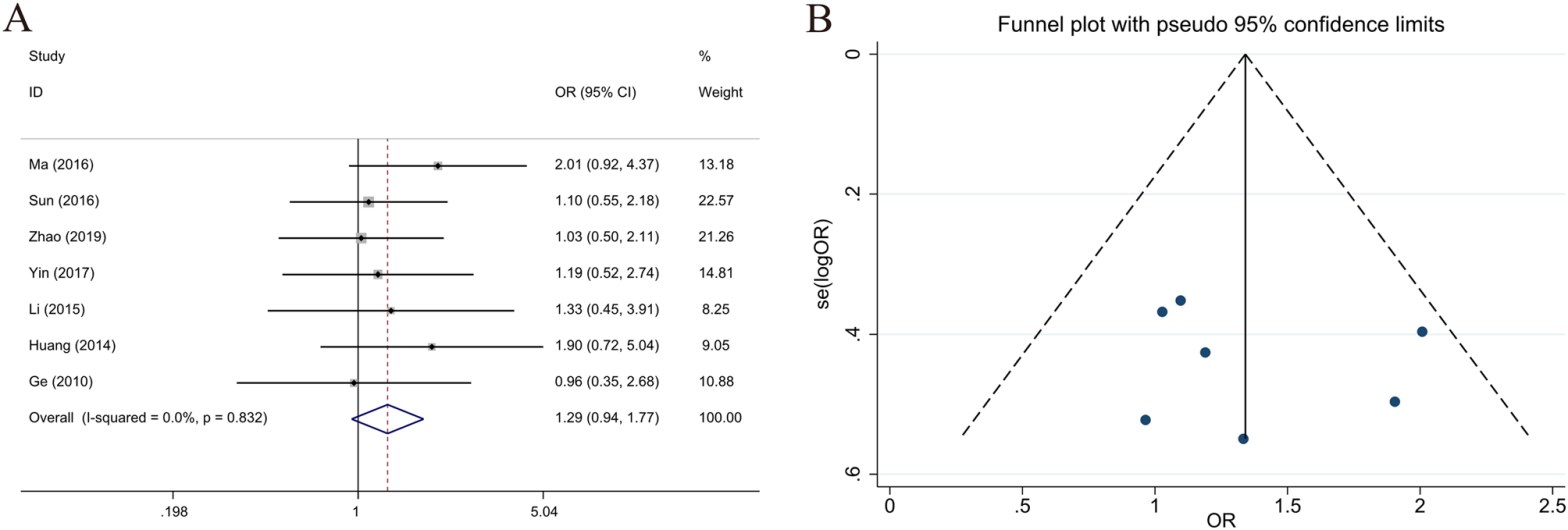
Forest plot (fixed-effects model) and funnel plot for publication bias test for the association between LKB1 expression and age (**A**,**B**) of patients with gastric cancer.

Data collected from seven studies revealed that 383 patients were older than 60, and 356 were younger than 60. Analysis results demonstrated that LKB1 expression was not correlated with age (OR 1.294; 95% CI 0.945–1.772; *P* = 0.108, I^2^ = 0.0%) (Fig. 3).

### Relationship between LKB1 expression and tumor size of gastric cancer

**Figure 4:**
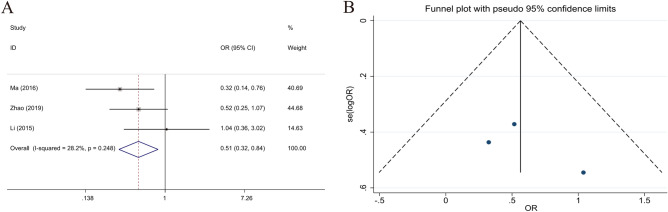
Forest plot (fixed-effects model) and funnel plot for publication bias test for the association between LKB1 expression and tumor size (**A**,**B**) of patients with gastric cancer.

Three studies with 299 patients reported tumor size (≥ 4 cm and < 4 cm). Statistical analysis results indicated that high LKB1 expression in GC was correlated with tumor size (≥ 4 cm vs. < 4 cm, OR 0.515; 95% CI 0.316–0.839; *P* = 0.008, I^2^ = 28.2%) (Fig. 4).

### Relationship between LKB1 expression and differentiation

**Figure 5:**
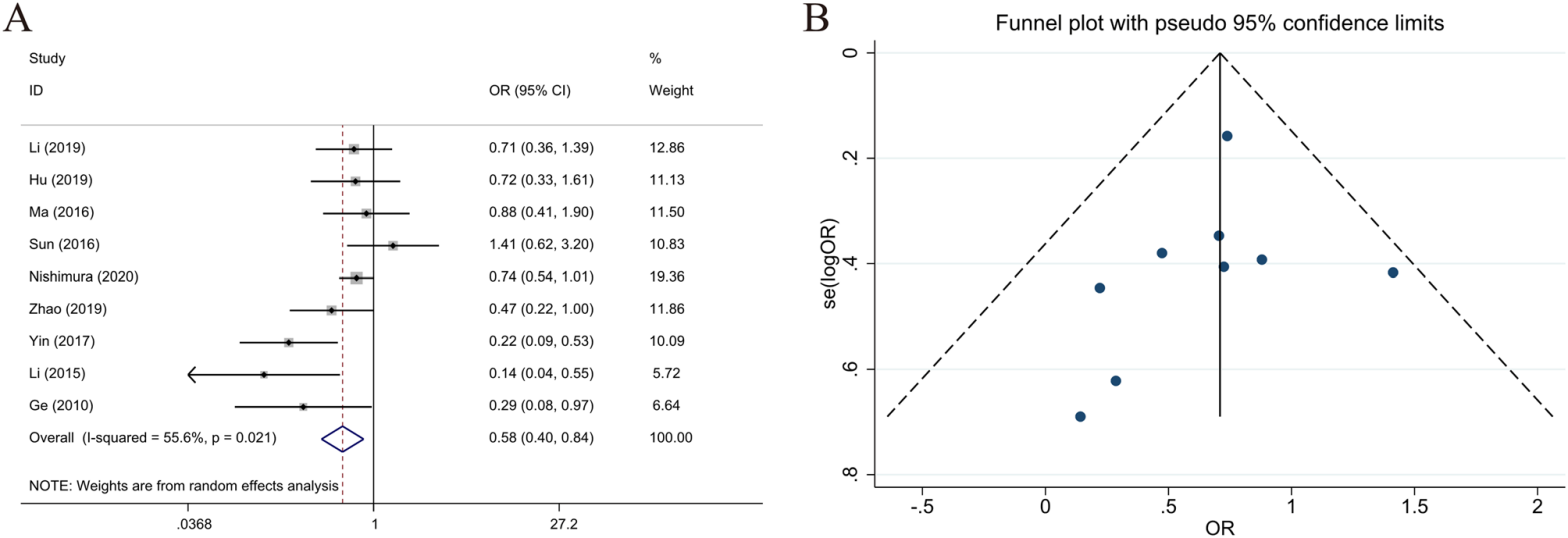
Forest plot (random-effects model) and funnel plot for publication bias test for the association between LKB1 expression and tumor differentiation (**A**,**B**) of patients with gastric cancer.

The nine selected studies with statistical data on differentiation poor-undifferentiated (n = 674) and well-moderate (n = 915) differentiation. The statistical results of LKB1 expression and differentiation indicated that high LKB1 expression correlates with the differentiation of GC (poor-undifferentiated vs. well-moderate, OR 0.643; 95% CI 0.521–0.794; *P* < 0.001, I^2^ = 55.6%). High LKB1 expression was a potential protective factor for the poor differentiation of GC (Fig. 5).

### Relationship between the LKB1 expression and the depth of invasion

**Figure 6:**
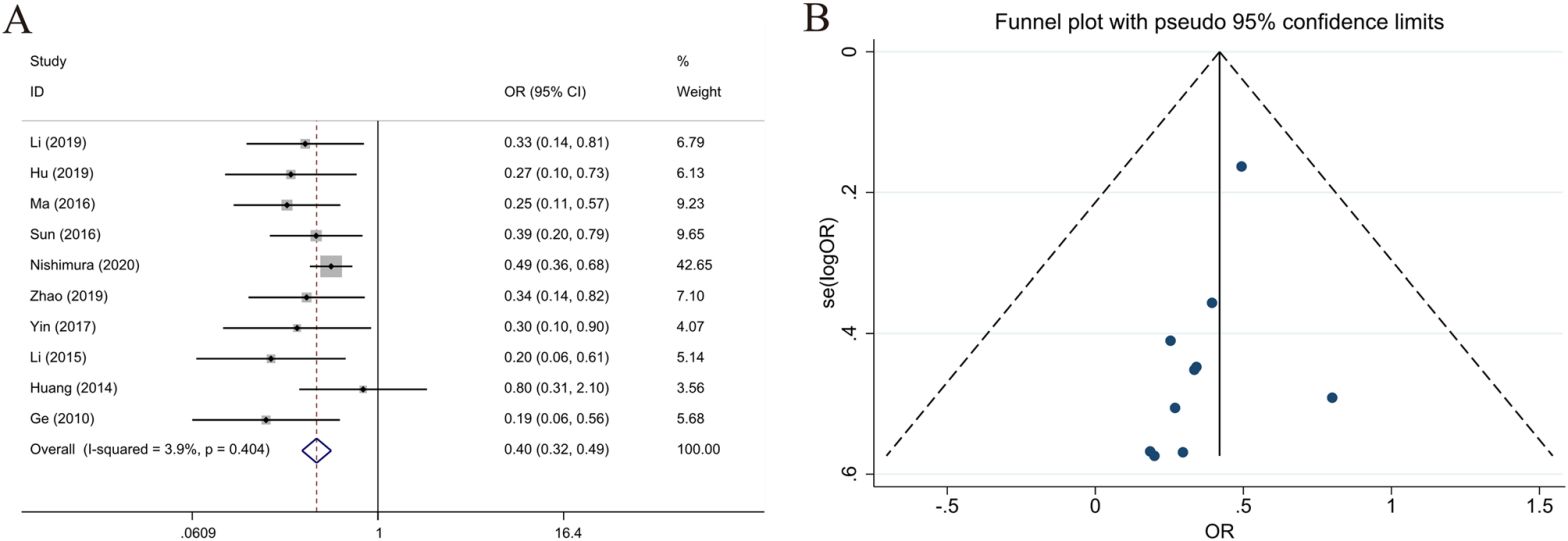
Forest plot (fixed-effects model) and funnel plot for publication bias test for the association between LKB1 expression and depth of invasion (**A**,**B**) of patients with gastric cancer.

Data on the depth of invasion were collected from 10 articles. The T1–T2 and T3–T4 groups included 604 and 1070 patients, respectively. The results indicated that high LKB1 expression correlated with the depth of pathological invasion (T3–T4 vs. T1–T2, OR 0.397; 95% CI 0.319–0.494; *P* < 0.001, I^2^ = 3.9%) (Fig. 6). High LKB1 expression was a protective factor for the depth of pathological invasion.

### Relationship between LKB1 expression and lymph node metastasis

**Figure 7:**
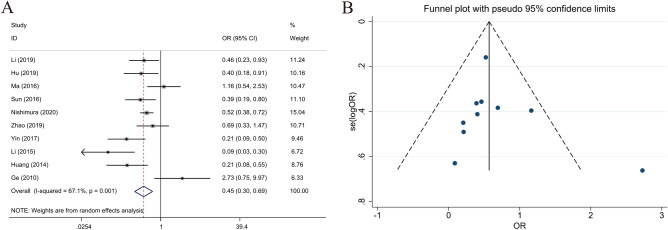
Forest plot (random-effects model) and Funnel plot for publication bias test for the association between LKB1 expression and lymph node invasion (**A**,**B**) of patients with gastric cancer.

Data from 10 studies reported the relationship between LKB1 expression and lymph node metastasis, including present (n = 969) and absent (n = 735). According to the analysis of LKB1 expression and lymph node metastasis, high LKB1 expression was associated with lymph node metastasis of GC (present vs. absent, OR 0.487; 95% CI 0.397–0.598; *P* = 0.01, I^2^ = 67.1%) (Fig. 7). Additionally, high LKB1 expression was a protective factor for lymph node metastasis in GC.

### Relationship between LKB1 expression and TNM stage

**Figure 8:**
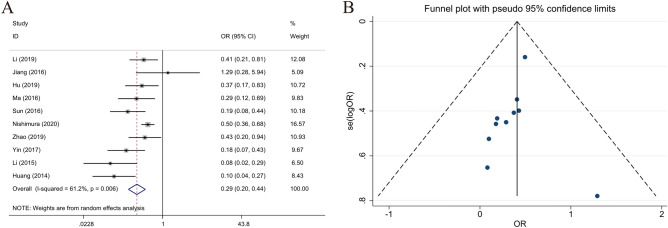
Forest plot (random-effects model) and funnel plot for publication bias test for the association between LKB1 expression and TNM stage (**A**,**B**) of patients with gastric cancer.

Among the 10 selected studies with data on the TNM stages of GC, the stage I/II and III/IV groups comprised 833 and 873 patients, respectively. According to the analysis of LKB1 expression and TNM stage, high LKB1 expression was related to the pathological TNM stage of GC (stage III/IV vs. stage I/II, OR 0.362; 95% CI 0.293–0.447; *P* = 0.006, I^2^ = 61.2%) (Fig. 8).

### Relationship between LKB1 expression and OS

**Figure 9:**
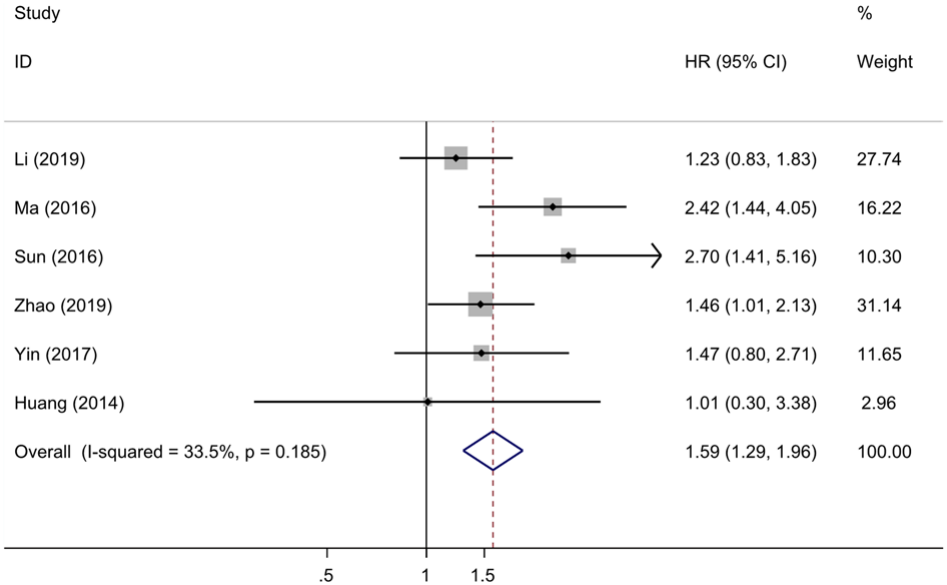
Forest plot (fixed-effects model) of the hazard ratio (HR) for the association between the low expression of LKB1 and overall survival (OS) of patients with gastric cancer.

Six studies explored the association between LKB1 expression and the OS of GC. The combined HR was used to evaluate the relationship between low expression of LKB1 and OS (HR = 1.59; 95% CI 1.29–1.96; *P* < 0.001, I^2^ = 33.5%). The results are shown in Fig. 9. The high LKB1 expression correlated with OS at 1-, 3- and 5-year OS. The results are shown in Table S2 and Figure S2. This meta-analysis demonstrated that low LKB1 expression is correlated to worse GC prognosis, and high LKB1 expression is related to good GC prognosis. LKB1 expression potentially predicts the prognosis of patients with GC.

### Sensitivity analysis and publication bias

**Figure 10:**
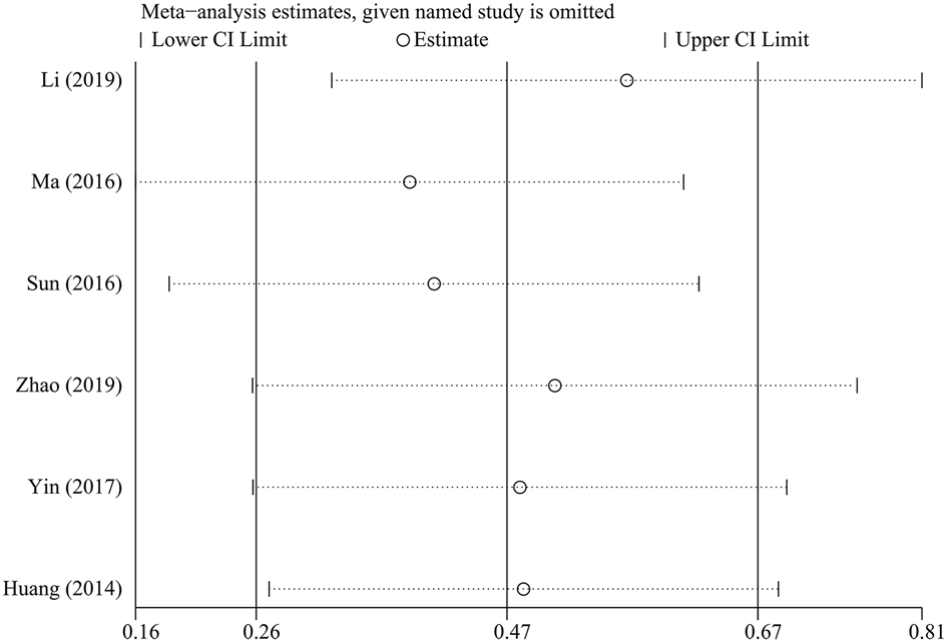
Sensitivity analysis of the association between LKB1 and overall survival (OS) of patients with gastric cancer.

**Figure 11:**
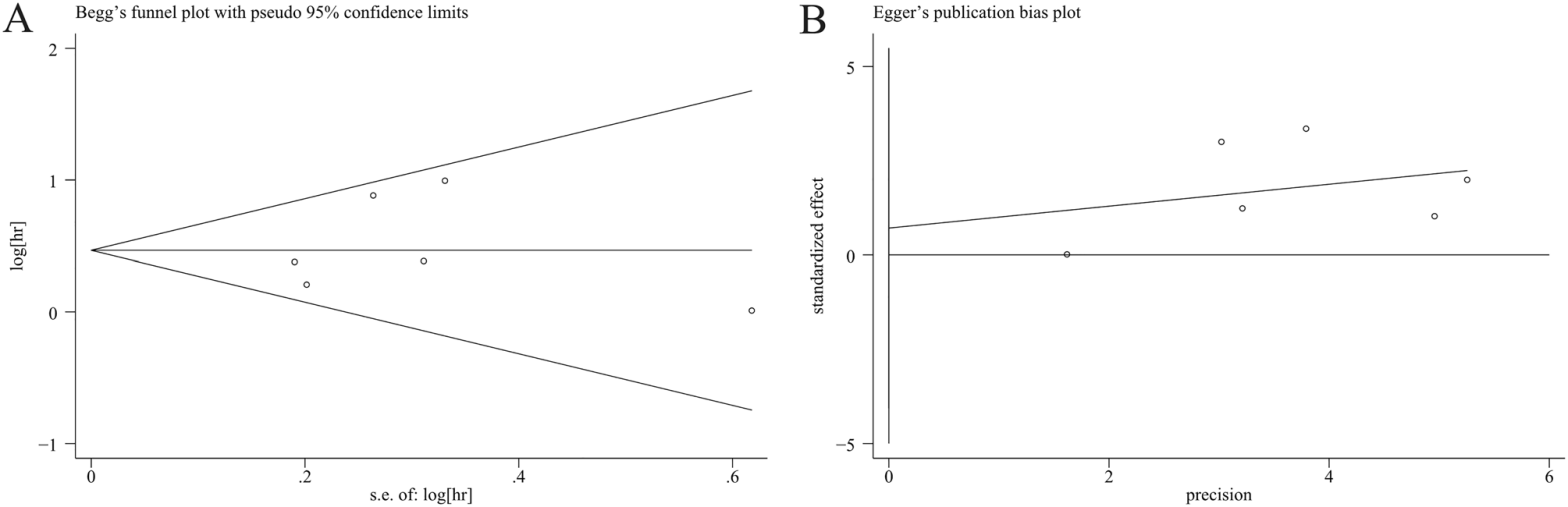
Funnel plots for detecting publication bias in terms of overall survival data. (**A**) Begg’s funnel plot using data of overall survival to detect publication bias; (**B**) Egger’s funnel plot using data of overall survival to detect publication bias.

We perform sensitivity analyses to determine the robustness of the OS-related results (Fig. 10). The result shows that no matter which single study was removed, the overall conclusion is stable, and no individual study dominated this meta-analysis. Moreover, Begg’s test and Egger’s test were performed to detect the publication bias, and the result of Begg’s test (*P* = 1.000) and Egger’s test (*P* = 0.700) implied there was no publication bias in our meta-analysis (Fig. 11).

## Discussion

GC is a global health problem, with one million new cases occurring annually^30^. Rapid advances in biotechnology have improved our understanding of the molecules and specific biomarkers associated with GC, facilitating new-drug discovery and novel diagnostic methods application^30^. However, GC’s survival rate has been very low over the past decades^31^.

LKB1 is an essential serine/threonine kinase that induces diverse cellular processes such as cell metabolism, cell proliferation, and cell migration^32^. LKB1 mutations or loss were widely found in different tumor types, such as cervical cancer^33^, ovarian cancer^34^, breast cancer^35,36^, pancreatic cancer^37^ and non-small-cell lung cancer(NSCLC)^38,39^. LKB1 also plays an important role in the tumor immune microenvironment, which is essential for tumor immune treatment^40^. Recently, many studies found that LKB1 plays a vital role in GC^15^. Previous studies indicated that low LKB1 expression was related to a significantly shorter OS and led to inferior therapeutic responsiveness to pembrolizumab in patients with GC, suggesting that LKB1 might be a potential immunotherapeutic target^41^. However, the relationship between LKB1 expression and clinicopathological parameters of GC patients was inconsistent across the different studies. A previous study has provided certain information regarding the prognostic value of LKB1 in patients with solid tumors. However, no meta-analysis has been made to evaluate the prognostic value of LKB1 expression in GC. Therefore, we performed the meta-analysis to investigate the relationship between LKB1 expression and the clinicopathological parameters and prognostic value of GC patients and found: (1) LKB1 expression is not associated with gender or age; (2) LKB1 expression is significantly correlated with tumor size, degree of differentiation, depth of invasion, lymph node metastasis, and TNM stage; (3) LKB1 expression is significantly correlated with OS; and LKB1 low expression is a risk factor for poor prognosis. Our findings indicate that LKB1 expression is a potential biomarker for predicting the survival prognosis of patients with GC.

While LKB1 expression is associated with GC^41^, Li et al. and Hu et al. studies demonstrated that high LKB1 expression is not associated with the gender and age of patients with GC^15,23^. Our meta-analysis found LKB1 expression is not associated with gender (*P* = 0.068) and age (*P* = 0.108) among patients with GC, consistent with previous studies.

Many conflicting results exist regarding LKB1 expression and clinicopathological parameters in patients with GC. First, Ma et al. suggested that high LKB1 expression is correlated with GC tumor size^16^. However, a study conducted by Zhao et al. revealed no statistically significant differences among the parameters^29^. Our meta-analysis results (*P* = 0.008) indicated that high LKB1 expression is associated with GC tumor size. Second, previous studies show a relationship between LKB1 expression and the degree of GC differentiation^25,28,29^. However, Li et al. believe that LKB1 expression is not associated with differentiation^23^. Our meta-analysis revealed that high LKB1 expression is associated with GC differentiation (*P* < 0.001). Third, several studies show high LKB1 expression is related to the depth of GC invasion^4,15,16,23^. However, a previous study suggests that high LKB1 expression is unrelated to the depth of GC invasion^26^. The data collected in our meta-analysis indicate that high LKB1 expression is a protective factor for the depth of invasion (*P* < 0.001). Fourth, some researchers believe that high LKB1 expression is related to lymph node metastasis in GC^4,15,16,23,26^. Nevertheless, others suggested that high LKB1 expression is unrelated to lymph node metastasis in GC^25,29^. According to our results (*P* < 0.001), it could be deduced that high LKB1 expression is a protective factor for lymph node metastasis in GC. Fifth, some studies have concluded that LKB1 expression is linked to TNM staging^3,15,16,23,29^, whereas others suggested that LKB1 expression is unrelated to the GC TNM stage^15,16,23,26,29^. The results of our meta-analysis indicate that high LKB1 expression is a protective factor for pathological TNM staging (*P* = 0.006). In summary, high LKB1 expression is associated with tumor size (*P* < 0.01), degree of differentiation (*P* < 0.001), depth of invasion (*P* < 0.001), lymph node metastasis (*P* = 0.01), and TNM stage (*P* < 0.01) of GC which reflected the ability of proliferation and metastasis of GC.

Several studies explore the relationship between LKB1 expression and OS of GC patients. A study conducted by Li et al. showed that high LKB1 expression is a favorable factor for OS and an independent prognostic marker in GC^23^. Decreased expression of LKB1 is associated with epithelial-mesenchymal transition and led to an unfavorable prognosis in GC^15^. LKB1 is reduced in GC and negatively correlated with p53 and surviving expression and plays an important role in predicting invasion and metastasis of GC^23^. Previous studies revealed that LKB1 acts as a critical regulator in various types of cancer through the AMPK/mTOR^42^, Wnt/β-catenin^43^, and Hippo signaling pathways^44,45^. Some studies have suggested that LKB1 loss promotes tumor proliferation by altering the NKX2-1/p53 pathway^46^ and that loss of the LKB1-AMPK signaling pathway is associated with prognosis in patients undergoing advanced non-small cell lung cancer chemotherapy^47^. Moreover, LKB1 loss expression promotes the nuclear translocation of Yap and β-catenin lead a poor prognosis of GC patients^16^. Our meta-analysis concluded that low LKB1 expression is a risk factor for the poor OS of GC patients; meanwhile, high LKB1 expression is an indicator of higher 1-, 3-, and 5-year OS rates. LKB1 expression may be an essential marker for predicting GC prognosis.

### Limitations

Certain limitations should be considered when interpreting this study’s results. First, we only involved the studies using IHC methods to detect LKB1; in situ hybridization (ISH), RT-qPCR, or ELISA also can detect LKB1. Further investigation should be taken to explore the expression of LKB1 detection in these methods in the future. Second, we only involve the study detection in tissue; the LKB1 expression in serum needs to be further studied. Third, although Begg’s and Egger’s tests revealed no publication bias, the literature selected for this meta-analysis was geographically limited, predominantly from East Asia. Fourth, the language types included in the study were restricted to Chinese and English, and articles published in other languages were excluded. Fifth, not all specific 95% CI values were directly extracted from the studies. Survival data were extracted from Kaplan–Meier curves. Besides, due to the unavailable of complete original data, we could not determine which time point or stage the LKB1 expression in gastric cancer performs best as a prognostic marker. Therefore, further research is required to evaluate the relationship between LKB1 expression and the clinicopathological features and prognosis of patients with GC.

## Conclusions

In conclusion, this meta-analysis suggests that LKB1 expression is significantly correlated with tumor size, differentiation, depth of invasion, lymph node metastasis, and TNM stage of GC. Low LKB1 expression is a risk factor for OS of GC, and high LKB1 expression is a protective factor for poor 1-, 3-, and 5-year OS of GC. LKB1 may be an important biomarker for clinical and predicting the prognosis of patients with GC.

## Supplementary Information

Supplementary Information.

## Data Availability

All data are available in the main text or the supplementary materials. The corresponding author (B.L.) had access to all the data in this study and took responsibility for the data's integrity and accuracy of data analysis.
