# Supplementary material for: Clinicopathological and prognostic significance of LKB1 expression in gastric cancer: a systematic review and meta-analysis
Source: Sci Rep. 2023 Jun 1;13:8937. doi: 10.1038/s41598-023-36239-5 (PMC10235053; doi:10.1038/s41598-023-36239-5)
Supplement: Supplementary file 1 — Supplementary Information. [file 41598_2023_36239_MOESM1_ESM.pdf]

Supplementary Information file

Clinicopathological and prognostic significance of LKB1 expression in gastric cancer: A systematic review and meta-analysis

Guojiang Tan[1+], Baiying Liu[1+*]

[1] Department of Gastrointestinal Surgery, the Third XiangYa Hospital of Central South University, China

[+]These authors share the first authorship on this work.

*Corresponding author

E-mail: liubaiyingdu@163.com

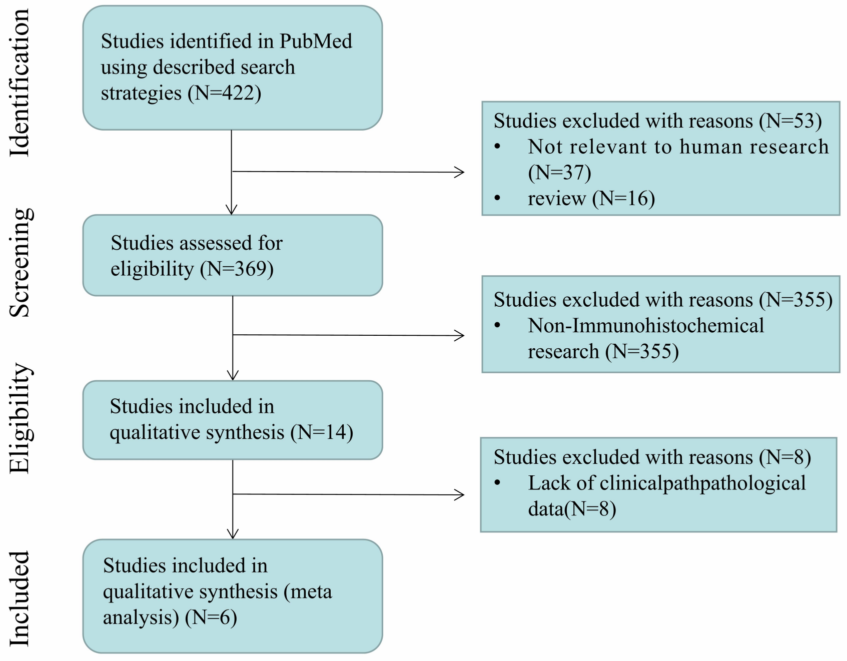

Figure S1. The details flowchart for selection in the PubMed database.

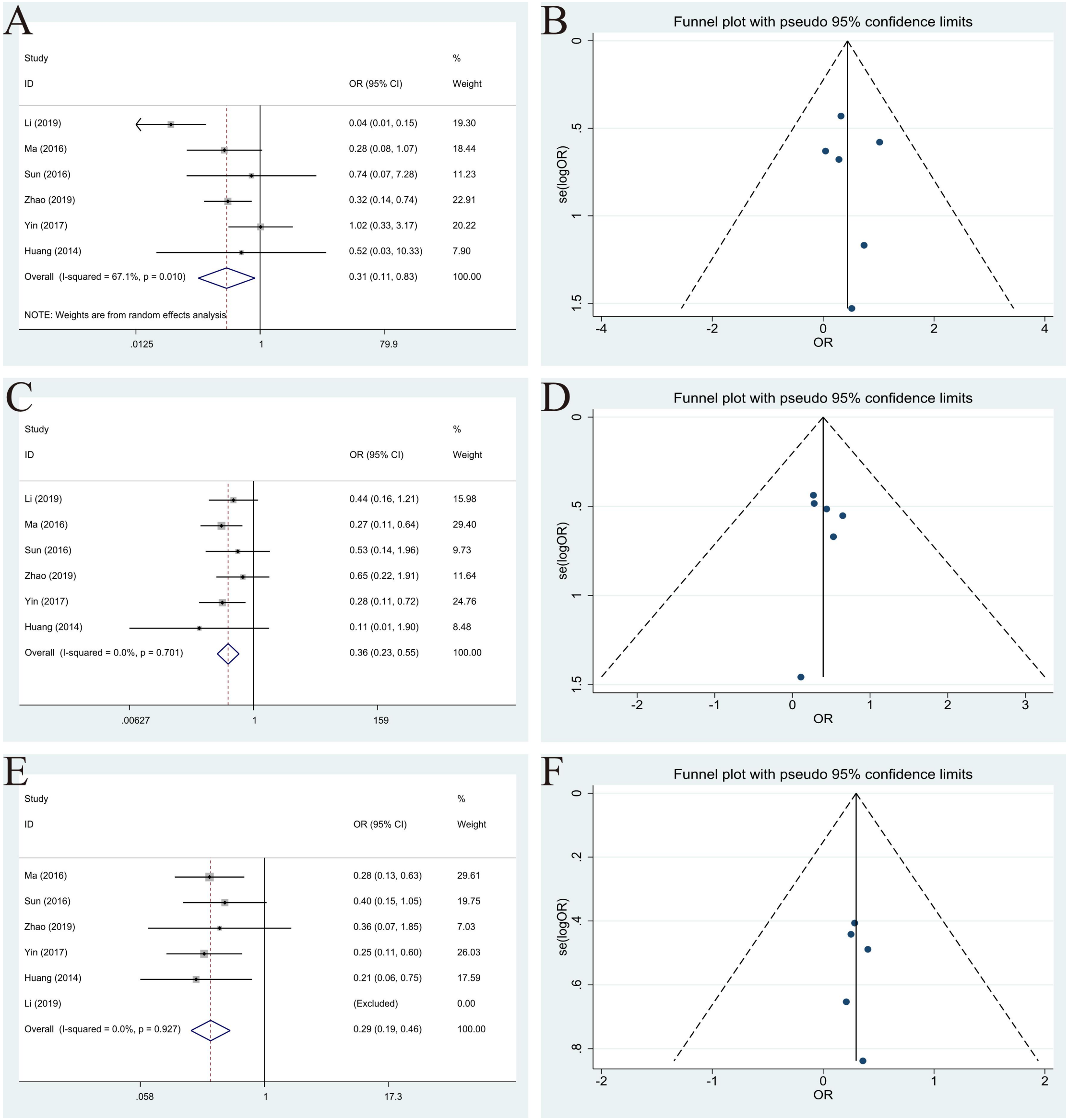

Figure S2. Forest plots and funnel plots for publication bias test for the association between high LKB1 expression and 1-year OS (A, B), 3-year OS (C, D), and 5-year OS (E, F) of patients with gastric cancer.

Quality criteria	Selection (4)				Comparability(2)		Exposure(3)			
	Case definition adequate (1)	Representativeness of the cases(1)	Selection of controls (1)	Definition of controls (1)	Comparability based on design or analysis(2)		Ascertainment of exposure(1)	Same method of ascertainment for cases and controls(1)	Non-Response rate(1)	Total (9)
Li/2019	●	●	●	●	●	●	○	●	●	8
Jiang/2016	●	●	●	●	●	●	●	●	●	9
Hu/2019	●	●	●	●	●	○	●	●	●	8
Ma/2016	●	●	●	●	○	●	●	●	○	7
Sun /2016	●	●	●	●	●	●	○	●	●	8
Nishimura/2020	●	●	●	○	●	●	●	○	●	7
Zhao/2019	●	●	●	●	●	●	●	●	○	8
Yin/2017	●	●	●	○	●	●	●	●	●	8
Li/2015	●	●	●	●	○	●	○	●	●	7
Huang/2014	●	●	●	○	●	●	●	●	●	8
Ge/2010	●	●	●	●	●	○	○	●	●	7

Table S1. The Newcastle-Ottawa scale(NOS) score of the eligible studies

Author (Year)	1 Year		3 Year		5 Year	
	OR(95%CI)	Weight(%)	OR(95%CI)	Weight(%)	OR(95%CI)	Weight(%)
Li (2019)	0.043 (0.013-0.148)	49.27	0.441 (0.161-1.209)	15.98	-	-
Ma (2016)	0.284 (0.075-1.072)	12.83	0.270 (0.115-0.637)	29.40	0.284 (0.128-0.630)	29.61
Sun (2016)	0.738 (0.075-7.278)	2.41	0.528 (0.142-1.964)	9.73	0.403 (0.155-1.050)	19.75
Zhao (2019)	0.321 (0.138-0.745)	25.74	0.648 (0.219-1.913)	11.64	0.358 (0.069-1.851)	7.03
Yin (2017)	1.019 (0.327-3.170)	7.80	0.280 (0.108-0.723)	24.76	0.251 (0.106-0.596)	26.03
Huang (2014)	0.516 (0.026-10.331)	1.94	0.109 (0.006-1.896)	8.48	0.208 (0.058-0.750)	17.59
overall	0.247 (0.152-0.404)	100.00	0.355 (0.228-0.553)	100.00	0.291 (0.185-0.456)	100.00

Table S2. Patient survival in relationship with expression of LKB1
